# Price Fairness, Consumer Attitude, and Loyalty in the U.S. Egg Market: The Moderating Roles of Tariff Concern and Education Level

**DOI:** 10.3390/foods14132243

**Published:** 2025-06-25

**Authors:** Min Gyung Kim, Joonho Moon

**Affiliations:** 1 College of Business Management, Hongik University, 2639, Sejong-ro, Jochiwon-eup, Sejong 30016, Republic of Korea; mkim@hongik.ac.kr; 2 Department of Tourism Administration, Kangwon National University, Chuncheon 24341, Republic of Korea

**Keywords:** egg, price fairness, attitude, loyalty, tariff concern, education level

## Abstract

The primary objective of this study is to investigate the relationships among perceived price fairness, consumer attitude, and loyalty in the context of egg consumption. In addition, the study examines the moderating effects of tariff-related concern and education level on these relationships. Data were collected through an online survey administered via the Clickworker platform, resulting in a sample of 311 U.S. consumers. The proposed hypotheses were tested using Hayes’s Process Macro (Model 7). The results reveal that both perceived price fairness and consumer attitude have significant positive effects on loyalty toward eggs. Moreover, a strong positive association was found between consumer attitude and loyalty. The analysis also identifies the moderating roles of tariff concern and education level, particularly in the relationship between price fairness and loyalty. This research contributes to the existing literature by offering insights into the dynamic interplay of five key variables in the context of consumer behavior within the egg market. Also, this research proposes practical implications focusing on various stakeholders.

## 1. Introduction

Eggs are a staple food consumed across diverse demographic groups due to their affordability and nutritional value [1,2,3]. As a basic commodity, eggs represent an essential good that is generally price-inelastic and influenced more by consumer perception than discretionary trends. Understanding consumer behavior in this market provides valuable insights into the dynamics of essential goods consumption and the broader patterns of sustainable consumer behavior. Egg prices are particularly susceptible to fluctuations arising from supply chain disruptions, disease outbreaks, and policy changes such as tariffs [4,5]. Given their necessity, consumers tend to respond strongly to such changes, especially with regard to perceived price fairness. Exploring consumer perceptions in this context can help identify critical thresholds of price tolerance, which may be essential for sustaining market stability. Prior research documents that eggs are among the most price-sensitive food items [6,7,8]. Consequently, perceived price fairness emerges as a key attribute for examining consumer behavior in the egg market, and this study positions it as a central construct.

In addition to price fairness, scholars addressed the significance of consumer attitude as a core determinant of behavior, noting that attitudes are typically formed over time and are relatively resistant to change [9,10]. Prior works claimed that eggs are a type of necessity good [2,3]. This characteristic implies that there may be a wide range of suppliers and intense competition in the market. In such a context, exploring loyalty becomes meaningful, as it implies a continued relationship with a specific supplier among many options [11,12]. Investigating consumer attitudes in this situation is also important, as attitudes are the result of long-term evaluations and have been actively inspected in the existing literature as a factor influencing loyalty [13,14]. Furthermore, consumer loyalty has been widely investigated as it directly contributes to sustained sales and brand equity. These conceptual frameworks are particularly relevant to egg consumption behavior [15,16]. Building on these insights, the present study focuses on perceived price fairness, consumer attitude, and loyalty as primary variables to investigate behavioral dynamics in the egg market.

The next domain of this work is the moderating roles of tariff concern and the education level of consumers. In 2025, the United States faced significant pressure from a dual crisis: a severe egg supply shortage due to avian influenza and newly implemented tariff policies [17,18]. In response, the U.S. government expanded egg imports, sourcing from countries such as Mexico, Brazil, Türkiye, and South Korea [17,19]. Scholars have argued that uncertainty in the supply of essential food items such as eggs can heighten consumer anxiety and increase sensitivity to price changes [20,21,22]. The government’s emergency strategy to rapidly increase egg imports further underscores the importance of understanding public reactions to such policies [18,19]. Despite the significance of this issue, little research has examined consumer perceptions related to egg tariffs. This study addresses this gap by exploring how consumer awareness of tariff policies influences their perception and behavior regarding eggs. Another focus of this work is to investigate the moderating role of education level in shaping consumer behavior. Previous research suggests that individuals with higher levels of education are more likely to understand contextual factors and respond to market stimuli in more informed ways [23,24]. This is likely because individuals with higher levels of education possess greater abilities to seek out and synthesize information in order to better understand situations. This framework can be extended to egg consumption behavior, where educational attainment may significantly influence how consumers perceive price fairness and respond to policy interventions such as tariffs.

All things considered, the first objective of this work is to examine consumer behavior in the egg market by focusing on key variables, including perceived price fairness, consumer attitude, and loyalty. As the second objective, this work scrutinizes the moderating effects of tariff awareness and educational level on the relationship between price fairness and consumer attitudes. The contribution of this study lies in its focus on price fairness as a central construct for understanding consumer behavior toward essential goods regarding attitude and loyalty as main attributes. By analyzing the moderating roles of tariff concern and education level, the research offers a more nuanced view of consumer behavior and delivers meaningful theoretical contributions to the literature on food consumption and pricing perception. From a practical standpoint, the findings of this study offer strategic implications for producers, retailers, and policymakers. Understanding consumer expectations around price fairness, product quality, and transparency in production processes can guide more effective market strategies. Furthermore, as consumer responses to economic policies like tariffs can have cascading effects across agricultural and retail sectors [25,26], insights from this study is likely to aid in anticipating behavioral shifts and mitigating negative market and public reactions.

## 2. Review of the Literature and Hypotheses Development

### 2.1. Price Fairness

Price fairness is commonly defined as consumers’ perception of whether a given price is justifiable, reasonable, or equitable [27,28]. Elevated or unexpected prices often deter purchase decisions, making price fairness a critical construct in consumer behavior research. Consequently, scholars have examined price fairness across various industry contexts to understand its broader implications. For example, Do et al. [27] investigated price fairness within logistics services, while Hride et al. [29] analyzed its impact on consumer behavior in electronic commerce. Similarly, studies by Sohaib et al. [30] and Singh et al. [31] explored the concept in the hotel and fast-food sectors, respectively. Halimi et al. [32] employed price fairness to account for consumer behavior in the domain of the food service business market. Collectively, these investigations underscore the pivotal role of perceived price fairness in shaping consumer decision-making across diverse markets.

### 2.2. Attitude

Attitude is defined as a long-term evaluative disposition toward a particular subject, and it is typically stable and resistant to change over time [10,33]. Owing to its enduring nature, attitude has been extensively examined in consumer behavior research as a key determinant of decision-making. For instance, Zaremohzzabieh et al. [34] investigated consumer attitudes toward green products, while Tsvakirai et al. [35] focused on attitudes toward cultured meat. Similarly, Dong and Gao [9] examined consumer attitudes in the context of wine consumption, and Szymkowiak et al. [36] explored attitudes related to food labeling and zero-waste initiatives. Sun and Moon [37] adopted attitude as the main attribute to examine consumers of bee products. These studies collectively highlight the centrality of attitude as a foundational construct in understanding and predicting consumer behavior across various domains.

### 2.3. Loyalty

Loyalty is commonly defined as the repeated purchase or patronage of preferred goods and services over time [16,38]. As a critical driver of long-term business success and sales growth, consumer loyalty has been a central focus in marketing and consumer behavior research [11,12]. For instance, Shyu et al. [39] examined the determinants of loyalty in the context of agricultural products, while Sun and Moon [40] analyzed restaurant consumer behavior using loyalty as the primary outcome variable. Similarly, Chen et al. [15] explored loyalty in the domain of tourist food consumption, and Cui et al. [41] investigated loyalty concerning fresh food purchases. Singh et al. [42] also emphasized the role of loyalty in shaping consumer behavior in the fast-food sector. Rabbanee et al. [43] explored consumers of genetically modified food using loyalty as a dependent variable. Collectively, these studies highlight the significance of loyalty as a key construct in understanding and predicting consumer decision-making across various contexts.

### 2.4. Hypotheses Development

In terms of loyalty, Martin et al. [44] identified a positive relationship between price fairness and consumer loyalty among retail customers. Hride et al. [29] further supported this relationship in the context of electronic commerce, revealing that fair pricing positively influences loyalty. Likewise, Konuk [28] found that price fairness exerts a positive impact on loyalty in the domain of brand management. Prum et al. [45] found a positive association between price fairness and loyalty by investigating hotel customers. Based on this body of literature, it can be inferred that perceived price fairness is likely to exert a positive influence on loyalty. Such a relationship is likely to appear in the case of the egg market. Accordingly, this study proposes the following research hypothesis:

**Hypothesis** **1:** *Price fairness positively affects loyalty*.

Sohaib et al. [30] found, in a study involving hotel customers, that perceived price fairness had a significant positive effect on consumer attitudes. Similarly, Park et al. [46] demonstrated a positive association between price fairness and attitude within the context of augmented product consumption. Khandelwal and Bajpai [47] also confirmed that attitude is positively influenced by price fairness, employing a linear regression analysis to support their findings. Halimi et al. [32] disclosed a positive effect of price fairness on consumer attitude in the domain of the food service market. It indicates that the positive relationship between price fairness and attitude is likely to appear in the domain of food consumers. The review of the literature implied that price fairness is likely to affect attitudes in the domain of the egg market. Therefore, this research proposes a hypothesis as follows:

**Hypothesis** **2:** *Price fairness positively affects attitude*.

The next focal point of this study is the relationship between consumer attitude and loyalty. Hussain et al. [14] found that a positive attitude significantly influenced loyalty among halal cosmetic consumers. Similarly, Albaity and Rahman [13] revealed that bank customers’ attitudes had a direct positive effect on their loyalty. Hwang et al. [48] also demonstrated that attitudes toward food technology significantly shape consumer loyalty. Lacap et al. [49] unveiled the positive influence of attitude on loyalty in the case of fast food customers. Rabbanee et al. [43] inspected genetically modified food consumers, and the results indicated that attitude exerted a positive effect on loyalty. From these findings collectively, it can be inferred that attitude can serve as a key determinant of loyalty across various consumption contexts. This relationship is likewise applicable to the egg market, where consumer attitudes may play a crucial role in shaping loyalty. Based on this review, the following research hypothesis is proposed:

**Hypothesis** **3:** *Attitude positively affects loyalty*.

### 2.5. Moderating Roles of Tariff Concern and Education Level

The previous literature documented that tariffs increase trade costs, impose greater burdens on consumers, and have the potential to trigger trade disputes, disrupt global supply chains, and contribute to inflationary pressures [50,51,52]. Indeed, previous studies have documented that tariff policies can lead to economic instability and uncertainty [51,52,53]. In such contexts, consumers may experience heightened anxiety due to the perceived risk of economic volatility associated with tariff implementation. Empirical findings also suggest that consumer behavior varies depending on the degree of uncertainty present in the environment [54,55]. Researchers additionally contended that tariffs influence consumer prices, leading consumers to exhibit price-related behaviors [56,57]. Taken together, this body of literature indicates that anxiety induced by tariff policies may lead to differential consumer responses. Thus, this research proposes a research hypothesis as follows:

**Hypothesis** **4:** *Tariff concern significantly moderates the relationship between price fairness and attitude*.

Previous works stated the importance of educational attainment in shaping individuals’ awareness of societal issues and their capacity to process complex information [58,59]. Scholars have also argued that individuals with higher levels of education tend to exhibit greater confidence in navigating challenging and uncertain conditions [23,24]. It can be inferred that more educated consumers are not only more engaged with social and economic concerns but may also be more resilient in the face of market disruptions. Scholars identified education as a significant moderating variable in shaping behavioral outcomes [60,61,62]. Drawing on these findings, it can be posited that egg consumers with higher educational attainment are better equipped to contextualize market fluctuations and are therefore less reactive to price changes. This may be attributed to their greater capacity for acquiring, processing, and evaluating market-related information. Collectively, this body of literature suggests that anxiety triggered by tariff policies may lead to heterogeneous consumer responses, depending on educational background. Building on these insights, it can be inferred that egg consumers with higher educational attainment are more likely to contextualize market fluctuations and respond less sensitively to price changes. Thus, the current work proposes the following research hypothesis:

**Hypothesis** **5:** *Education level significantly moderates the relationship between price fairness and attitude*.

## 3. Method

### 3.1. Research Model

**Figure 1:**
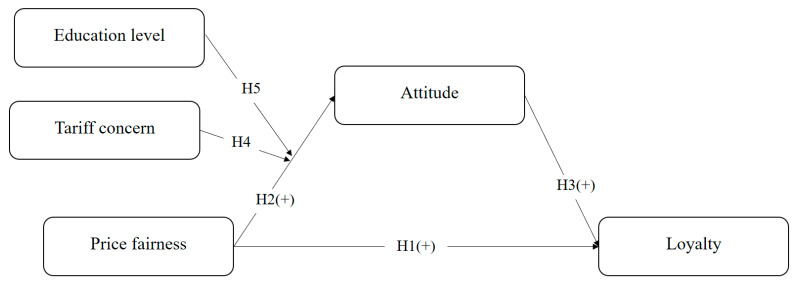
Research model.

Figure 1 illustrates the research model using five attributes: price fairness (independent variable), attitude (mediator), loyalty (dependent variable), tariff concern (moderating variable), and education level (moderating variable). It shows that price fairness has a positive impact on both attitude and loyalty. Attitude is positively associated with loyalty. Moreover, this research proposed the significant moderating roles of tariff concern and education level on the effect of price fairness on attitude.

### 3.2. Measurement Illustration

Table 1 illustrates the measurement items. A five-point Likert scale (1 = strongly disagree, 5 = strongly agree) was used to measure the main items. Only attitude was measured by a five-point semantic differential scale (e.g., 1 = bad, 5 = good). Three attributes, including price fairness, attitude, and loyalty, were measured by referencing prior works. To develop the measurement of tariff concerns, we consulted with two experts in consumer behavior research. Price fairness was operationally defined as the extent to which consumers perceived the presented price of eggs as appropriate. Attitude refers to consumers’ perceptions shaped by long-term, accumulated evaluations of eggs. Loyalty was defined as the intention of consumers to consistently purchase a specific type of egg. Lastly, tariff concern was conceptualized as the anxiety experienced by consumers regarding potential economic losses resulting from the imposition of tariffs. All variables were measured by four items, other than education level. The measurement of education level was measured as an ordinal variable (1 = less than college, 3 = more than a graduate degree).

### 3.3. Data Analysis

This study conducted a frequency analysis to obtain demographic information of the survey respondents. To evaluate convergent validity, confirmatory factor analysis was performed. By prior research, convergent validity was assessed using multiple criteria: standardized factor loadings greater than 0.50, average variance extracted (AVE) exceeding 0.50, and construct reliability (CR) above 0.7 [63,64,65]. Subsequently, the study computed the mean values and standard deviations for each construct. Discriminant validity was assessed using a correlation matrix based on the criterion that the square root of the AVE for each construct should exceed the corresponding inter-construct correlation coefficients [63,64,65]. To evaluate the goodness-of-fit of the measurement model, several fit indices were employed, including χ^2^/df < 3, root mean square residual (RMR) < 0.1, and additional indices such as the goodness-of-fit index (GFI), normed fit index (NFI), relative fit index (RFI), incremental fit index (IFI), Tucker–Lewis index (TLI), and comparative fit index (CFI), all with recommended thresholds above 0.80. The root mean square error of approximation (RMSEA) was also used, with values less than 0.10 indicating acceptable model fit [63,64,65]. This work adopted analysis of moment structure (AMOS) to perform confirmatory factor analysis. Furthermore, to test the research hypotheses, the study employed Hayes’s Process Macro (Model 7) with 5000 bootstrap samples to ensure the robustness and reliability of the mediation and moderation analyses because prior studies addressed that the analytic instrument is adequate to explore the moderating and mediating effects [66,67]. This research additionally used the simple slope method to scrutinize the moderating effects of tariff concern and education level. The simple slope analysis offers more than a mere confirmation of the significance of a moderation effect; it enables researchers to precisely identify the conditions under which the effect intensifies or diminishes [67]. Moreover, by visualizing the results through interaction plots, this method facilitates clearer interpretation and enhances accessibility for non-experts [67].

### 3.4. Data Collection

This research collected the data using Clickworker (https://clickworker.com, accessed on 11 April 2025). Numerous works adopted the platform to collect data in the social science quantitative research domain [68,69]. Such previous works could become the clue for the assurance of the data quality for statistical inference. The period of the data collection was between 11 and 13 April in 2025. The period is after the declaration of the tariff policy of the U.S. The participants were U.S. consumers because U.S. consumers are affected by the policy of tariffs in the domain of egg consumption during the data collection period. This study employed random sampling to enhance the objectivity of the results and provided respondents with guidelines to answer the survey based on their egg purchasing experience within the past two weeks. The number of observations is 311, which might be adequate to implement statistical analysis including validity and reliability tests and regression analysis because the rule of thumb states that a single measurement item requires 10 observations to reach credible statistical inference [65].

Table 2 presents the demographic information of the survey participants. The numbers of males and females are 93 and 218, respectively. Regarding age, the 30s and 40s accounted for approximately 71.5 percent of survey participants. Regarding the monthly household income, approximately 63 percent of respondents had a monthly household income below USD 5000. Table 2 also presents the information of education level (less than college: 138, bachelor’s: 115, and more than graduate degree: 58) and weekly consumption amount of egg (less than 1: 40, 2–4: 182, 5–8: 68, and more than 9: 21).

## 4. Results of Empirical Analysis

### 4.1. Results of Confirmatory Factor Analysis and Convergent Validity

Table 3 shows the results of the confirmatory factor analysis. The indices of goodness of fit indicated that the results of confirmatory factor analysis are statistically significant (χ^2^ = 271.787, χ^2^/df = 2.773, RMR = 0.064, GFI = 0.896, NFI = 0.938, RFI = 0.924, IFI = 0.959, TLI = 0.950, CFI = 0.959, and RMSEA = 0.076). All factor loadings, CRs, and AVEs are greater than the cut-off values. Based on the values, the convergent validity of measurement items was ensured. Table 3 also presents the mean values of four attributes: price fairness (mean = 2.53, SD = 1.24), attitude (mean = 4.26, SD = 0.88), loyalty (mean = 3.92, SD = 0.97), and tariff concern (mean = 3.92, SD = 1.05).

### 4.2. Correlation Matrix for Discriminant Validity

Table 4 exhibits the correlation matrix. Comparing diagonal and off-diagonal values, the discriminant validity of all attributes was ensured. Loyalty positively correlates with attitude (r = 0.396, *p* < 0.05) and price fairness (r = 0.189, *p* < 0.05). Attitude positively correlates with price fairness (r = 0.152, *p* < 0.05), and price fairness positively correlates with tariff concern (r = 0.130, *p* < 0.05).

### 4.3. Results of Hypotheses Testing

Table 5 exhibits the results of the hypotheses testing using Hayes’ Process Macro, Model 7. Three models are statistically significant, given the *p*-values of F-statistics (*p* < 0.05). Price fairness positively affects both attitude (β = 0.403, *p*< 0.05) and loyalty (β = 0.103, *p*< 0.05). Also, attitude is positively associated with loyalty (β = 0.410, *p*< 0.05). Price fairness × Tariff concern (β = 0.101, *p*< 0.05) and Price fairness × Education level (β = −0.174, *p*< 0.05) exerted significant effects on attitude. All in all, all the proposed hypotheses are supported.

**Figure 2:**
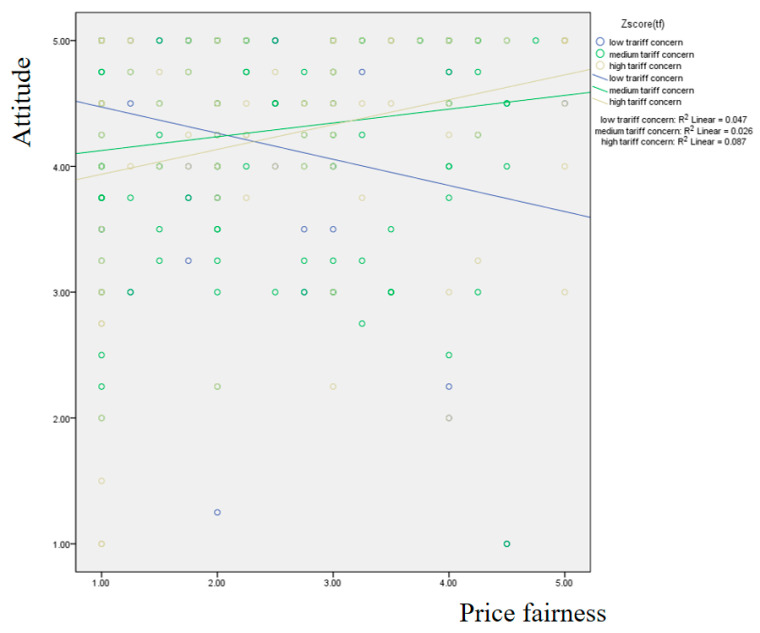
Results of the simple slope method focusing on the moderating effect of tariff concern.

Figure 2 illustrates the results of the simple slope method. The medium (β = 0.102, *p* < 0.05) and high (β = 0.204, *p* < 0.05) tariff concern groups showed the significant effect of price fairness on attitude. That is, the high tariff concern group was more sensitive to the effect of price fairness on attitude to eggs.

**Figure 3:**
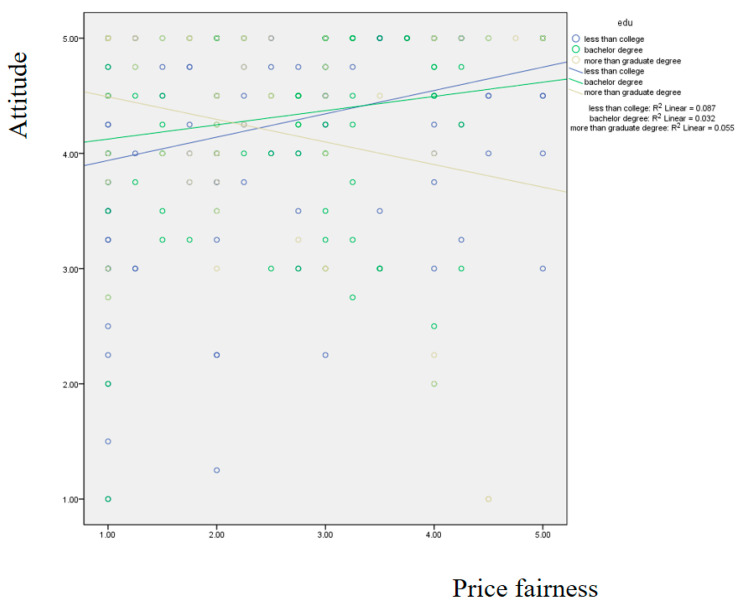
Results of the simple slope method focusing on the moderating effect of education level.

Figure 3 depicts the moderating effect of education level using the simple slope method. The results showed that only the low education level group showed a significant impact of price fairness on attitude (β = 0.229, *p*< 0.05).

## 5. Discussion

This study investigated the relationships among perceived price fairness, consumer attitudes, and loyalty within the egg market. A secondary objective was to examine the moderating effects of tariff concerns and educational attainment on these relationships, specifically within the context of egg consumers in the United States—a market significantly impacted by global tariff dynamics. The analysis revealed that consumers’ perceptions of price fairness regarding eggs were relatively low (mean = 2.53), implying that during the data collection period, price perceptions may have been negatively influenced by prevailing market conditions. The analysis revealed that the average attitude toward eggs was relatively high (mean = 4.26), suggesting that consumers hold generally positive perceptions of eggs. This favorable evaluation likely stems from the widespread recognition of eggs as a key nutritional food source, closely linked to personal health and well-being. These findings indicate that eggs are regarded by consumers as an essential component of the diet. Furthermore, the results show that consumers expressed a considerable level of concern regarding tariff-related issues, as reflected in a relatively high mean score of 3.92.

Empirical results demonstrated that perceived price fairness had a significant positive effect on both consumer attitudes and loyalty. Moreover, consumer attitudes appeared to positively influence loyalty, underscoring their mediating role in fostering long-term consumer commitment within the egg market. These findings suggest that the price of eggs plays a critical role in shaping consumers’ positive attitudes and loyalty. This implies that eggs function as a type of necessity good, with price exerting a significant influence on consumer decision-making and perceptions.

With respect to tariff concerns, the findings indicated that consumers experiencing higher levels of anxiety related to tariffs responded more sensitively to perceptions of price fairness. Specifically, these individuals exhibited more favorable attitudes when egg prices were perceived as stable, suggesting that price stability serves as a psychological buffer against tariff-induced uncertainty. It can be inferred that consumers’ perceptions of eggs as a necessity good may lead to varying effects of price attitudes influenced by concerns over tariffs. In other words, anxiety regarding tariffs can be inferred to act as a key factor shaping consumer behavior. The study also identified a significant moderating effect of education level on the relationship between price fairness and consumer attitudes. Consumers with lower educational attainment were more reactive to perceptions of price fairness, whereas those with higher education levels demonstrated a greater capacity to contextualize price fluctuations within broader structural and economic frameworks. This suggests that higher educational attainment may mitigate anxiety related to price volatility by enhancing consumers’ understanding of market dynamics. Conversely, individuals with limited access to or understanding of such information may experience elevated sensitivity to pricing concerns—a pattern supported by the data.

## 6. Conclusions

### 6.1. Theoretical Implications

This study makes several notable theoretical contributions. First, it provides valuable insights into consumer behavior in the egg market by integrating the constructs of perceived price fairness, consumer attitude, and loyalty—dimensions that have received limited attention in the context of basic commodities. Second, the research advances the literature by examining consumer perceptions within the framework of U.S. tariff policies, a relatively underexplored area. Specifically, the identification of tariff concern as a moderating variable offers a novel understanding of how macroeconomic policies influence individual consumption behavior in essential food markets. Third, the finding that consumers with lower levels of educational attainment are more sensitive to perceived price fairness adds a socio-demographic dimension to consumer segmentation, enriching the discourse on how structural factors shape market responses. Fourth, by confirming the positive influence of perceived price fairness on both consumer attitude [30,70] and loyalty [30,47], this study enhances the external validity of previous research. Additionally, it contributes to the broader literature on price perception and consumer decision-making by affirming the mediating role of attitude in the relationship between fairness perceptions and loyalty. The observed consistency with prior studies on the relationship between attitude and loyalty in the context of egg consumption [14,48] further strengthens the theoretical significance of these constructs in food markets.

### 6.2. Practical Implications

The findings offer several practical implications for policymakers, retailers, and producers. From a governmental perspective, it is essential to secure stable supply chains before implementing tariff policies, as consumer anxiety and price sensitivity may increase under uncertain market conditions. Policymakers also might be able to consider the broader behavioral consequences of tariffs and communicate effectively with the public to mitigate negative sentiment. For sellers, the results documented that consumer attitudes and loyalty were closely tied to perceptions of price fairness. Therefore, maintaining consistent pricing through reliable supplier relationships might become a viable strategy to enhance brand trust and customer retention. Producers, in turn, may benefit from increased transparency in production processes, particularly in promoting quality standards and health-oriented messaging, which could reinforce positive consumer attitudes and loyalty. Finally, proactive public communication strategies during periods of price fluctuation, such as using mass media to explain the causes of rising egg prices, could help maintain consumer trust and reduce speculation or misinformation.

### 6.3. Limitations and Future Research Directions

Despite its contributions, this study has several limitations. First, the scope of the research was limited to the U.S. market and focused solely on the consumer perspective. Future studies could incorporate insights from the supply side, including producers, retailers, and importers, to offer a more holistic view of the egg value chain. Second, the present study employed only price fairness as the independent variable. Future research could examine a broader range of factors, such as perceived quality, ethical sourcing, and brand image, to provide a more comprehensive understanding of consumer behavior. Third, the study relied exclusively on survey data. Future work could employ mixed methods approaches, such as experiments, interviews, or longitudinal data, to triangulate findings and enhance methodological robustness.

## Figures and Tables

**Table 1:** Illustration of measurement.

Attribute	Code	Measurement Item	Reference
Price fairness	PF1	Egg prices were fair.	Konuk [28] Hride et al. [29]
	PF2	Egg prices were reasonable.	
	PF3	Egg prices were appropriate.	
	PF4	Egg prices were acceptable.	
Attitude	AT1	For me, the egg is (negative–positive)	Hussain et al. [14] Sun & Moon [37]
	AT2	For me, the egg is (bad–good)	
	AT3	For me, the egg is (useless–useful)	
	AT4	For me, the egg is (unfavorable–favorable)	
Loyalty	LY1	I am going to use the same eggs again.	Chen et al. [15] Hwang et al. [48]
	LY2	I will purchase the same eggs.	
	LY3	I am willing to pay for the same eggs.	
	LY4	I am willing to buy the same eggs.	
Tariff concern	TF1	Tariffs will cause economic anxiety.	Developed through consulting
	TF2	Tariffs will increase economic uncertainty.	
	TF3	Tariffs will destabilize the economy.	
	TF4	Tariffs will make the economic situation unpredictable.	

**Table 2:** Information on survey participants (N = 311).

Item	Frequency	Percentage
Male	93	29.9
Female	218	70.1
20s	40	12.9
30s	119	38.3
40s	103	33.1
50s	38	12.2
Older than 60	11	3.5
Monthly household income		
Under USD 2500	91	29.3
USD 2500 and USD 4999	105	33.8
USD 5000 and USD 7499	35	11.3
USD 7500 and USD 9999	22	7.1
Over USD 10,000	58	18.6
Education level		
Less than college	138	44.4
Bachelor’s degree	115	37.0
More than a graduate degree	58	18.6
Weekly consumption amount of eggs		
Less than 1 egg	40	12.9
2~4 eggs	182	58.5
5~8 eggs	68	21.9
More than 9 eggs	21	6.8

**Table 3:** Confirmatory factor analysis.

Construct	Code	Loading	Mean (SD)	CR	AVE
Price fairness	PF1	0.893	2.53 (1.24)	0.957	0.849
	PF2	0.965			
	PF3	0.923			
	PF4	0.903			
Attitude	AT1	0.815	4.26 (0.88)	0.893	0.676
	AT2	0.897			
	AT3	0.758			
	AT4	0.815			
Loyalty	LY1	0.693	3.92 (0.97)	0.895	0.683
	LY2	0.823			
	LY3	0.837			
	LY4	0.936			
Tariff concern	TF1	0.846	3.92 (1.05)	0.940	0.796
	TF2	0.909			
	TF3	0.897			
	TF4	0.917			

Note: SD stands for standard deviation; goodness of fit indices: χ^2^ = 271.787, df = 98, χ^2^/df = 2.773, RMR = 0.064, GFI = 0.896, NFI = 0.938, RFI = 0.924, IFI = 0.959, TLI = 0.950, CFI = 0.959, RMSEA = 0.076; CR stands for construct reliability; AVE is average variance extracted.

**Table 4:** Correlation matrix results.

Variable	1	2	3	4
1. Loyalty	0.826			
2. Attitude	0.396 *	0.822		
3. Price fairness	0.189 *	0.152 *	0.921	
4. Tariff concern	−0.056	0.037	0.130 *	0.892

Note: Diagonal is the square root of the average variance extracted (AVE), * *p* < 0.05.

**Table 5:** Hypothesis testing results using Hayes’ Process Macro, Model 7.

	Model 1 Attitude		Model 2 Attitude		Model 3 Loyalty	
	β	t Value	β	t Value	β	t Value
Constant	4.888	11.72 *	3.262	12.00 *	1.912	7.48 *
Price fairness	−0.304	−1.88	0.403	4.12 *	0.103	2.52 *
Tariff concern	−0.224	−2.18 *				
Education level			0.432	2.96 *		
Price fairness × Tariff concern	0.101	2.62 *				
Price fairness × Education level			−0.174	−3.30 *		
Attitude					0.410	7.16 *
F value	4.81 *		6.14 *		334.89 *	
R^2^	0.0449		0.0566		0.6843	
Conditional effect of focal predictor						
Tariff concern						
3.00	0.009	0.01				
4.00	0.102	2.55 *				
5.00	0.204	3.73 *				
Education level						
1.00			0.229	4.25 *		
2.00			0.054	1.28		
3.00			−0.119	−1.50		
Index of mediated moderation	Index	LLCI ULCI	Index	LLCI ULCI		
	0.041 *	0.0059 0.0834	−0.071 *	−0.128 −0.019		

Note: * *p* < 0.05. LLCI: Lower Level Confidence Interval. ULCI: Upper Level Confidence Interval.

## Data Availability

The data presented in this study are available upon request from the corresponding author. The data are not publicly available due to privacy concerns.
